# Progressive Pernicious Anæmia

**Published:** 1875-05

**Authors:** 


### PROGRESSIVE PERNICIOUS ANÆMIA.

Under this name, Biermer has recently collected fifteen cases of a peculiar disease which does not appear to have been previously placed in any pathological system. The most prominent symptom of the disease is an increasing paleness of the integumentary surfaces, together with the usual phenomena of anæmia. The anæmia increases, and the patients, almost without exception, soon die. The *post-mortem* section reveals an extraordinary scarcity of blood in all parts of the body, though there is no corresponding loss of fat tissue, or other sign of emaciation.

The form of anæmia differs from others in—1. The absence of any sufficient etiological cause. 2. The extraordinary degree of blood-impoverishment and the connection of the anæmic symptoms with certain changes in the circulatory apparatus. 3. The appearance of febrile movements which have no anatomical basis. 4. The progressive character of the anæmia and its extremely malignant course, which cannot generally be checked by theraputical means. Evidently this disease does not occur frequently, or it would have

19

been described before. The greater number of cases appear to have occurred in Zurich and Basle, Switzerland. Most of the patients were women between twenty and forty years of age, and pregnancy appears to have had some connection with its cause. In other cases, in both sexes, the development of the disease appears to be more dependent on bad living, troubles of digestion, diarrhœa, etc. From the extreme degree of the anæmia, with the very slight atrophy and emancipation, it would seem that the probable cause of the disease is of such a nature as to exert a very considerable effect on the blood formation, while it affects but slightly the other functions of the organism.

Gusserow's observations have shown that transfusion is of no use in these cases. Only one of Biermer's cases left the hospital somewhat improved ; the others died, as was also the case with the five pregnant women mentioned by Gusserow. In severe symptomatic anæmia, on the contrary, the patients usually recover with proper treatment. Biermer and Gusserow did not find the contracted condition of the aortic system which is common in anæmia. The blood is pale, thin, and but slightly coagulable. In on case Ponfick found an almost entire absence of fibrin. Œdema becomes marked toward the close of the disease. The red blood-corpuscles were diminished in number; the number of the white blood-corpuscles was usually, though not always, increased. The spleen, lymphatic glands, and marrow of the bones, were unaffected. Biermer almost constantly found the pulse weakened and undulating, with a high systolic secondary sound of the heart and in the large vessels, although no valvular lesion was found in the *post-mortems.* An hemorrhage diathesis was usually developed early, with bleeding from the nose and gums, and sometimes extravasations under the skin and small cerebral hemorrhages. In all of Biermer's and in one of Immermann's cases there were retinal hemorrhages, but these did not in all cases cause disturbance of vision. The changes in the circulatory apparatus consisted in a partial fatty degeneration of muscular tisue of the heart. There was also fatty degeneration of the renal epithelium, hepatic cells, and the gastric glands. The fever seldom reaches a high degree, is varying, and frequently there are intermissions of considerable length. Treatment does not seem to be of any use.—Immermann in *Archiv f. klin Med.*, and *Uegeskrift f. Laeger*, July, 1874.—*G. R. C., in New York Medical Journal.*

